# Robot Helps When Robot Fits: Examining the Role of Baby Robots in Fertility Promotion

**DOI:** 10.3390/healthcare7040147

**Published:** 2019-11-15

**Authors:** Yao Song, Zhenzhen Qin, Tao Kang, Yang Jin

**Affiliations:** 1 School of Design, The Hong Kong Polytechnic University, Hung Hom, Hong Kong 00852, China; yao.song@connect.polyu.hk; 2 School of Journalism and Communication, Anhui Normal University, Wuhu 241002, China; 3 Wuhu Media Group, Wuhu 241001, China; Whrb_1@163.com; 4 General Affairs Department, Anhui College of Traditional Chinese Medicine, Wuhu 241002, China; jinyang@ahzyygz.edu.cn

**Keywords:** fertility intention, social robot, China, subjective norms, perceived behavioral control

## Abstract

Considering China is facing a precipitous decline in its population, there is an emerging trend of developing baby robots to encourage people’s willingness to become “parents”. Based on the decomposed theory of planned behavior and the theory of uncanny valley, this study empirically investigated whether a baby robot could perform as a prominent antecedent of fertility intention in China, and how this relates to its visual appearance. Consistent with prior research, the current study used a between-subjects design to show (1) a baby robot could significantly improve people’s fertility attitude through temporal visual stimulation; (2) fertility attitude, subjective norms from peers, and perceived behavioral control of finance could significantly contribute to fertility intention. Theoretical contributions and implications are discussed in this study.

## 1. Introduction

A falling birth rate could lead to widespread social and economic problems. Like many countries in the post fertility transition, China is facing a precipitous decline in its population, setting the stage shortly for potential demographic and economic issues. According to a recent report by the Chinese Academy of Social Sciences [1], China’s total fertility rate has been below 1.6 since 1996, which is lower than the replacement level fertility rate of 2.1. After a brief uptick from 2013 to 2016, the birth rate fell again in 2017, with 17.2 million babies born compared to 17.9 in 2016. Although the Chinese government has recognized the worrisome demographic trend and in 2015 abolished the one-child policy (replaced by a two-child policy) [2], the overall number of births continued to drop. It has been estimated that the total Chinese population will present negative growth around 2028, though others believe it would come sooner or has already begun [1].

Declining populations are creating many social problems in countries where this phenomenon is occurring [3,4,5]. To address this issue, some scholars and practitioners have exerted efforts to understand the mechanism of fertility promotion globally [6,7,8,9,10]. Smart technology drives healthcare and social development more than any other force [11,12,13]. For instance, robot-assisted therapy has been developed in the care of older adults with dementia [14]. Matarić et al., [15] showed robot coaches effectively aid stroke patient rehabilitation by providing social and physical assistance. Recently, a trend for developing baby robots has emerged as a means of encouraging people’s willingness to become “parents.” It has been suggested that a baby robot affording the simulating experience of mothering and fathering is effective in influencing the perceptions regarding the significance of having a child and parenting behavior [16]. The practice of developing baby robots, however, is implemented with both hopes and questions [17,18]. Whether it improves the willingness of parenting or not is uncertain for empirical researchers.

The attempt of baby robots as infant simulators for fertility education can be traced to the early pilot program of virtual infant parenting [19]. It was initially aimed to prevent teenage pregnancy in Australia and the US, nevertheless, controversial effects have been reported. For instance, a recent longitudinal experiment showed that the teenage girls who have access to an infant simulator were more likely to encourage childbearing than the control group [18]. Besides, some studies revealed an insignificant difference between the student group with infant simulators and the group without the simulators [19]. Nevertheless, the design-related factors, such as the visual appearance of infant simulators, have been much neglected in previous research investigating the consequent effects of virtual infant parenting. As relevant evidence revealed in robot research, visual appearance is influential in settings where it is preferred to have the user expect humanlike performance from the robot [20,21,22,23]. Besides, more recent research suggests the phenomenon of “Uncanny Valley”(UV) in designing the visual appearance of humanoid robots [24]. Its consistency in the application of baby robots is ambiguous.

Based on the well-defined theory of planned behavior in fertility research [25], this study aims to investigate whether a baby robot performs as a prominent antecedent of fertility intention in China, and how this relates to its visual appearance. Our empirical observation as a design guideline can enrich the current literature on fertility promotion from an emerging perspective of baby robots.

## 2. Literature Overview

### 2.1. Baby Robots and Parenting Promotion

China is not alone in confronting a shrinking population. It has been reported that nearly half of the world’s population now live in countries with fertility rates at or below replacement level, and nearly all countries will reach low fertility levels in the next two decades [3,4,5]. The long-term low fertility rate will lead to a high level of aging and population decline, which will bring multiple socio- economic challenges [26]. Therefore, relevant research is being conducted in countries with very low fertility to support and encourage women’s willingness for fertility from multiple perspectives, such as social environment [6,7], religious belief [8], and family-friendly public policy [9,10]. Previous attention has been mainly driven by the importance of women’s fertility attitudes while recent research has revealed the prominent role of men in shaping fertility behavior [27].

Among these, there is an increasingly growing interest in the infant simulator-based approach to promoting fertility intention [28,29]. Various humanoid robots have been designed to stimulate a human infant’s physical behaviors. These interactive means and experiences attempt to provide couples an opportunity to experience “parenting” [30,31]. Toyota launched Kirobo Mini, for instance, which can recognize and respond to people in a high-pitched tone while being unstable in its movements. Prospective parents can care for a robotic baby while deciding whether they want a real baby of their own [16]. Nevertheless, the empirical observation of whether baby robots improve parenting is limited in current research.

Prior research has reported a controversial relationship between infant simulators and teenage pregnancy behavior. The theory of fertility and infant simulators dates back to the Virtual Infant Parenting (VIP) program aiming at preventing teenage pregnancy in Australia and the US, and often referred to as “Baby Think It Over” [19]. Scholars argue that this well-designed simulation experience is useful in influencing the perceptions regarding the significance of having a child and fertility behavior in the future [32]. A longitudinal experiment conducted in Australia, however, indicated that access to infant simulators was more likely to encourage teenage girls’ childbearing than the control group [33]. Relevant research also reported an insignificant difference between the student group with infant simulators and the group without the simulators [34]. The educational value of infant simulators in influencing fertility behavior needs to be confirmed further.

### 2.2. Humanoid Robot Design and the Uncanny Valley

To address the complex users’ reactions to robots, scholars argue that the visual appearance of humanoid robots is effective in influencing users’ attitudes towards an interactive system and predicting consequent behavioral response [20,21,22]. The presence of a humanoid face as a robot head can result in an efficient, engaging, and social interaction between humans and robots [35]. This is advantageous in settings where it is preferred to have the user expected humanlike performance from the robot, such as a robot portraying a standardized human infant [23]. Relevant empirical observations report that humanoid facial displays are more attractive and motivate users’ interest more than other presences [36]. The reason lies in the fact that the human face works with emotional and conversational signals encoded as facial displays [37]. The humanoid level of a robot head improves the perception of humanness; consequently, it encourages users’ cognitive attitudes towards it [38]. However, if a mismatch occurs between the realism of facial appearance and robot character, it may have an adverse effect on the acceptance of that [39].

More recent research has revealed that negative reactions to imperfect humanoid robots may be caused by the well-known phenomenon of UV proposed by Mori [24]. It is a psychological theory about the effect that involving human feelings keep changing as the robot is made to look more human-like. Mori’s graph of the uncanny valley is widely used to enhance the design of humanoid robots and 3D computer-animated characters. Despite its dominance, the existence of UV is controversial [40,41]. Most studies aiming to address the issue are conducted in the contexts of humanoid robots primarily for general social tasks [20,23], the accompaniment of baby robots has seldom been investigated based on the theory of UV.

Baby robots to enhance parenthood experience strongly rely on their humanness and lifeness. The facial presence on a baby robot is assumed to be a crucial component of the overall visual appearance. However, the current design application is various without a clear guideline to indicate its relative effects on users’ responses. Therefore, incorporating the theory of UV and baby robot design is significant in revealing whether the humanoid appearance can influence users’ attitudinal reactions and the consequent decision of having a child.

### 2.3. Fertility Intention and the Planned Behavior

Much of the previous research into fertility promotion is underpinned by the theory of fertility intention [42,43], also known as childbearing intention and parenting intention. Behind its assumption is that having a child is the result of a reasoned decision. With this view, a large number of studies discussed fertility intention by deeming low fertility intention as equivalent to low fertility behavior, and vice versa [44,45,46]. For example, Zheng et al. [47] conducted a large-scale survey in China to examine the effects of government policy and economic and social development on fertility intention. Their results reveal an insignificant relationship between government control and the low fertility in Jiangsu province.

In recent years, however, the inconsistency between fertility intention and actual behavior has gained much attention [42]. Schoen et al. [48] examined 2812 non-Hispanic Whites; the results indicate that fertility intention does contribute additional predictive power. A similar observation is made by another study [49]. Instead, Toulemon and Testa [50] argue that the relationship between fertility intention and actual fertility behavior is quite loose because it depends on many other non-subjective factors. To answer this theoretical gap, Bongaarts [51,52] construes that the inconsistency between fertility intention and parenting behavior―actual fertility less than desired family size―is caused by an increasing average age of childbearing, involuntary infecundity, and the competition effect. Based on Bongaarts’ theory, scholars investigated the fertility decline in China [53].

More recent research suggests a psychological perspective to explain the path from fertility intention to parenting behavior [25]. Proposed by Ajzen [42], the theory of planned behavior (TPB) illustrates that fertility behavior is affected and resulted by cognitive evaluation. Briefly, according to TPB, the intention to have or not to have a child is determined by three kinds of factors: (1) attitude towards the fertility, which refers to the degree to which a person has a favorable or unfavorable evaluation of the question of having a child or not; (2) subjective norms, which refer to the perceived social pressure to have a child or not; and (3) perceived behavior control, which refers to perceived presence of factors that can influence an individual’s ability to have a child.

Though TPB has been applied in understanding the psychological path to fertility behavior in the Chinese context, the prominent antecedents of the three factors predicting fertility intention are limited in previous studies. Luo and Mao [25], for instance, confirmed that attitude towards fertility, subjective norms, and behavior control could influence the fertility intention and behavior of women who are qualified to have two children. The latest research has extended the model and proposed the decomposed theory of planned behavior [54,55], in which subjective norms are sub-divided into norms from peers and norms from family while perceived behavioral control are sub-divided into behavioral control of finance and control of energy. Thus, it leaves questions of how to improve fertility intention from these factors.

**Figure 1:**
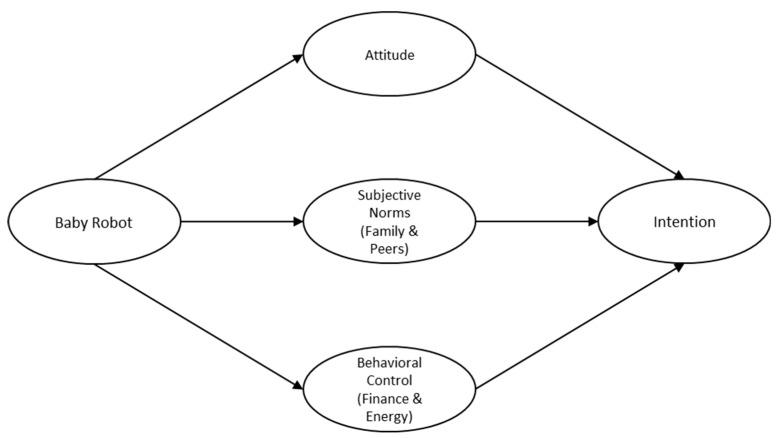
The theoretical model of the current study.

As the aforementioned functions of baby robots in improving the willingness of having a child, this study assumes that an individual’s fertility intention is improved by the accompaniment of a baby robot following a psychological path of decomposed TPB (see Figure 1). The results will provide preliminary evidence of baby robots as an influential antecedent to promote fertility intention within the Chinese context.

## 3. Research Method

### 3.1. Measurement Items

Considering the theory of planned behavior [56,57], we used a nine-point Likert scale to measure fertility attitude, subjective norms, and perceived behavioral control. All the measurement items were retrieved from the prior relevant research [25,54,55,58,59]. To specify, fertility attitude was measured by ‘‘I would consider the bond between husband and wife when deciding to have a child’’ (1 = Strongly Disagree, 9 = Strongly Agree) [25]. Consistent with literature in Section 2, subjective norms were measured from two perspectives, peers and family, through a two-items Likert scale (‘‘I would consider the opinions of my relatives when deciding to have a child/The opinions of my friends could influence my decision on childbearing’’, 1 = Strongly Disagree, 9 = Strongly Agree) [54,55]. Perceived behavioral control was also measured from two perspectives, finance and physical energy (‘‘I feel I have the energy to handle taking care of a child/I feel I can get enough financial resources to raise a child”; 1 = Strongly Disagree, 9 = Strongly Agree) [54,55]. Fertility intention was measured by the difference between the ideal and actual number of children in the survey [25], where 0 referred to the actual number of children they had was equal or greater than they intended to have while 1 referred to the actual number of children they have was less than they intended to have. Considering the research was conducted in the context of China, we adopted a professional translation service to translate the original English items into Chinese and then translated them back into English, ensuring consistency and accuracy. Table 1 shows the measurement items and relevant definitions from previous literature [25].

### 3.2. Experiment Stimuli

**Figure 2:**
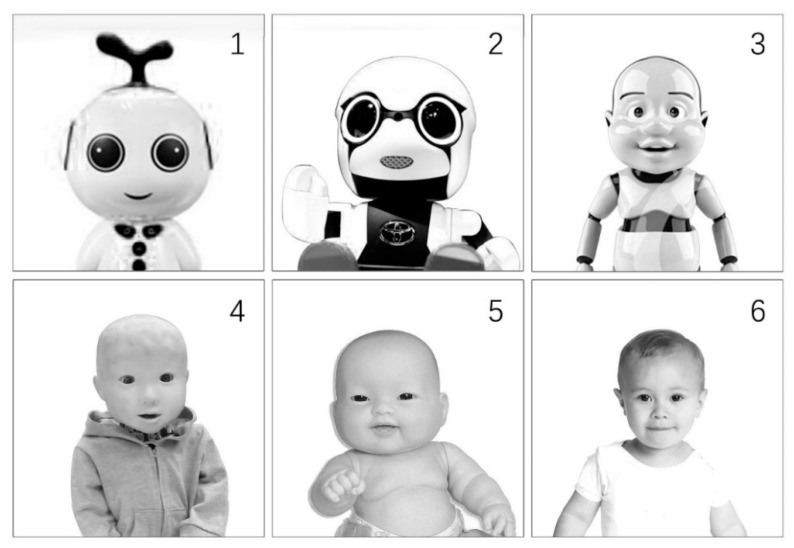
The baby robot stimuli utilized in this study.

Although there are not many infant simulators in our daily lives, the robot stimuli used in the current study were still adapted by a professional designer from the real infant robots in the current market. For example, No.2 infant robot was adapted from Toyota’s Kirobo Mini, which aimed to encourage couples to become “parents” [17,60]. No.4 robot was adapted from a child robot of Osaka University [61]. No.6 robot was adapted from a real infant picture, which was presumed to be an infant robot with a maximum degree of human resemblance. Besides, a panel of three robotics researchers was invited to sort the robots in the extent of resemblance to a human being. Accordingly, No.1 robot was believed to be the robot with the least human features; No.6 robot was thought to be the robot with most human features; No.3 and No.4 robot were rated as the robots with medium human features, imperfect human-resemblance features. Furthermore, the potential confounding elements, such as background, proportions, face direction, eye gaze, and gender, were all controlled the same in the six stimuli. Regarding this, this study used a between-subjects experimental design with these six different robot stimuli (No.1–No.6; see Figure 2).

### 3.3. Experiment Procedure

In order to explore the relationship among robot stimuli to the cognitive intention of fertility, we recruited a sample group from an anonymous Chinese “survey monkey”, Wen Juan Xing, to collect data. Wen Juan Xing is a reliable survey collection resource, specializing in recruiting Chinese participants and a large number of studies in the literature of Chinese social science have been conducted via this platform (similar to Amazon Mechanical Turk) [62,63,64]. This platform contains around 3 million users who come from every region of China with various occupations, and the gender proportion is approximately equal [64]. Considering the geographical distance and population diversity in China, we adopted Wen Juan Xing as a data collection tool in the current study.

The reference number of the ethical approval for research involving human subjects in Anhui College of Traditional Chinese Medicine is HSERS2019050001. We recruited a total of 180 subjects in this study. To be more specific, the participants were initially exposed to our task. After acknowledging the purpose of this study and the screening criteria, they could decide whether to participate in this study. The selection criteria were that they should be in the status of either being married, divorced, separated or widowed. Table 2 shows the detailed demographic information. Considering the distribution of the difference between the ideal and actual number of children, the result suggested 57.2% participants’ (N = 103; fertility intention = 0) ideal number of children is equal or higher than their actual number of children while 42.7% participants (N = 77; fertility intention = 1) ideal of number of children is less than their actual number of children (see Table 3).

**Figure 3:**
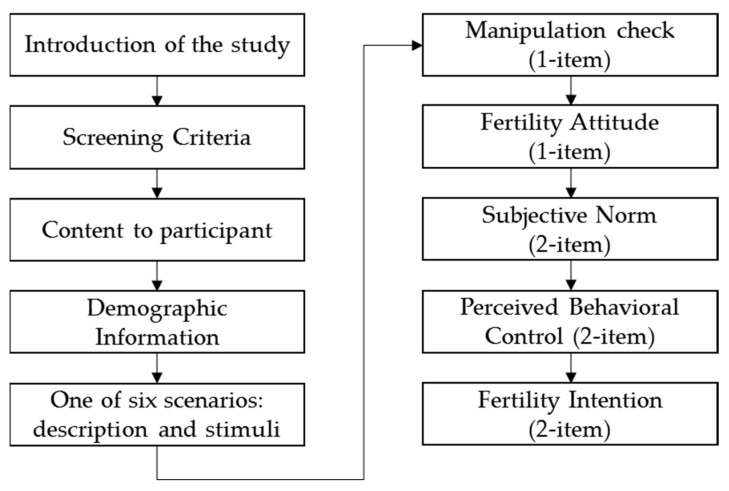
The experimental procedure in this study.

As for the experiment procedure, participants first met the screening criteria and consented to take part in this survey. Then they needed to fill in the demographic information and were informed that they were going to evaluate a baby robot. Each robot stimulus was assigned with 30 participants and they were all asked to look at the stimulus (a baby robot) and to consider taking care of it. After that, they were required to answer a few questions and a manipulation check (the degree of human- appearance resemblance they thought of the robot; 1 = strongly disagree; 9 = strongly agree). Figure 3 shows the experimental procedure.

## 4. Findings

MANOVA and Logit Regression were performed to analyze the data through SPSS 22.0. There were no missing or incomplete responses in the data set. Consistent with our predicted extent of human resemblance in the robot stimuli, manipulation check confirmed the extent of human resemblance gradually increased from No.1 robot to No.6 robot (Mean = 2.50, 2.57, 2.70, 3.53, 4.53, 7.00, respectively; SD = 1.78, 2.39, 2.20, 2.66, 3.11, 2.46, respectively). ANOVA analysis showed a significant difference in the human resemblance between No.1 and No.5/6 (both *p* < 0.05), suggesting experiment manipulation was successful.

As for the main analysis, a MANOVA analysis on fertility attitude, subjective norms and perceived behavioral control was conducted. Table 4 shows the means, SD (standard deviations), and Pearson correlations of different factors.

**Figure 4:**
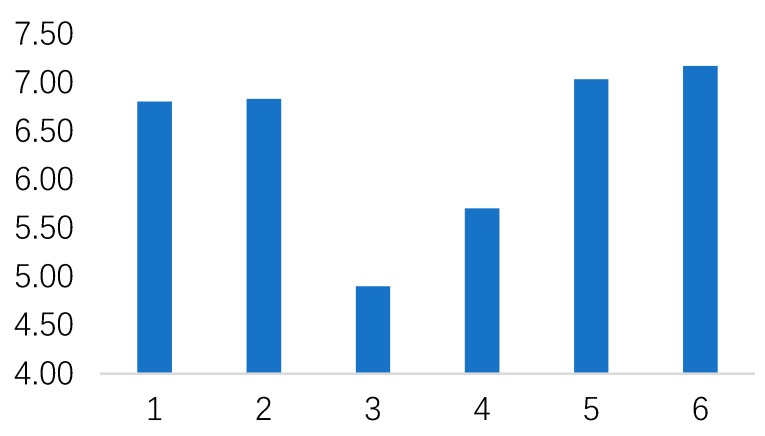
The relationship between fertility attitude under different stimuli.

With respect to fertility attitude, one-way ANOVA results suggested a significant difference between different stimuli (Mean for No.1–No.6 robot = 6.80 vs 6.83 vs 4.90 vs 5.70 vs 7.03 vs 7.17, respectively; SD for No.1–No.6 robot = 2.06 vs 2.55 vs 2.92 vs 2.26 vs 1.77 vs 1.64, respectively; F (5, 174) = 4.863; *p* <0.05; see Figure 4 and Table 5). Consistent with the theory of UV [23,41,65], people’s fertility intentions did not follow a linear trend. Instead, it dropped suddenly when the level of the robot’s human-resemblance increased to a certain level (No.3). Then, it gradually increased as the level of robot’s human-resemblance kept increasing (No.4/5/6). The post-hoc analysis showed people tended to have a significantly lower fertility attitude in No.3 robot compared to No.1/2/5/6 robot.

**Figure 5:**
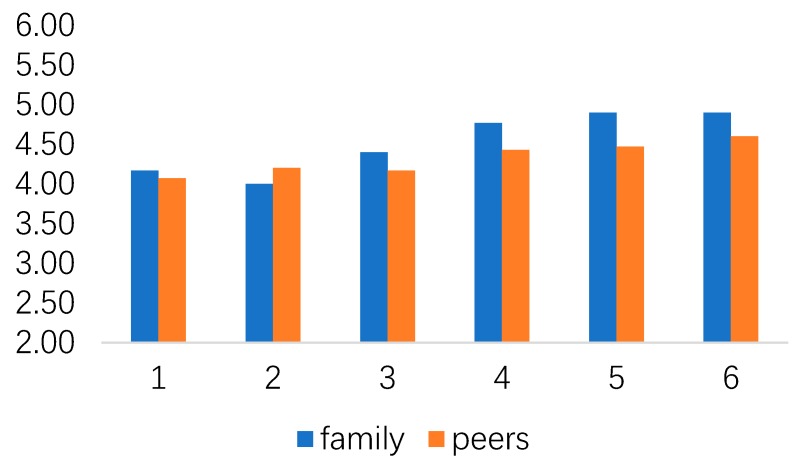
The relationship between subjective norms under different stimuli.

**Figure 6:**
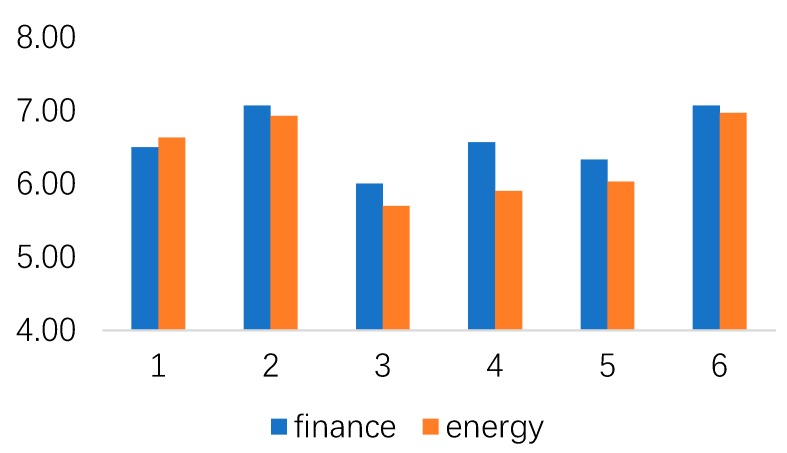
The relationship between perceived behavioral control under different stimuli.

Moreover, different robot stimuli did not significantly influence people’s subjective norm (for family: F (5, 174) = 0.730, ns; and, for peers: F (5, 174) = 0.200, ns) and perceived behavioral control (for finance: F (5, 174) = 1.277, ns; for energy: F (5, 174) = 2.225, ns). Figure 5 and Figure 6 show people’s subjective norms and perceived behavioral control under different stimuli.

As for the fertility intention, a binary logistic regression was introduced to explore the factors that contribute to fertility intention. According to the theory of planned behavior, an empirical analysis was further developed to investigate the effect of fertility attitude, subjective norms (family and peers), and perceived behavioral control (finance and energy).

Table 6 shows the result of this regression. The logistic regression was statistically significant (*chi*-square (8) = 31.445; *p* <0.05). The current model accounted for 21.5% of the variance in fertility intention with 68.9% identification accuracy. According to the result, fertility attitude was positively associated with fertility intention. While subjective norms from peers significantly contributed to fertility intention, subjective norms from family did not have a significant impact on fertility intention. Similarly, while perceived behavioral control of finance was positively related to fertility intention, behavioral control of energy only showed a marginal influence on fertility intention. In addition, age worked as a significantly negative indicator of fertility intention. Men showed somehow greater fertility intention compared with women however the effect was only marginal. Besides, no other significant effects were found.

## 5. Conclusions

Given the ambiguous relationship between the visual appearance of baby robots and people’s fertility intention, this paper introduced a behavioral experiment approach and tried to obtain a deeper understanding of the effect of a baby robot on people’s fertility behavior. Based on the decomposed theory of planned behavior, results show that the visual appearance of a baby robot could increase people’s fertility attitude. Under a temporal visual stimulation, the uncanny valley effects in baby robot design are associated with people’s fertility attitude, in which the degree of human-resemblance in a baby robot and fertility attitude followed a U-shape relationship. In other words, people tend to have a temporally higher fertility attitude when facing the baby robot with the most or the least human features, compared with the baby robot with the medium human features. However, the temporal visual effects of baby robots do not significantly contribute to the subjective norms (family and peers) and perceived behavioral control (finance and energy). This result in the current study is consistent with prior research: the temporal visual reaction towards stimuli would be more associated with individual cognition and attitude formation, rather than with long-term social cognition and control beliefs [66,67]. Subjective norms are the cultural products of acceptable group conducts [68,69], indeed, they are formed in the context of society and culture, representing informal knowledge that governs the conduct of members in the society [70]. Perceived behavioral control is a subjective assessment of easiness and difficulty in the performance of behavior [57], which is hardly determined by visual stimuli [55].

Regarding the relationship between fertility attitude, subjective norms, perceived behavioral control, and fertility intention, results show that fertility attitude is a significant indicator of promoting fertility intention. This is consistent with the prior research [25,44,58]. Besides, based on the decomposed theory of planned behavior [54,55], this study subdivides the subjective norms and perceived behavioral control into two sub-constructs, family and peers, and finance and energy. Results indicate that, in the context of the Chinese population, subjective norms from peers is significantly associated with fertility attitude while subjective norms from family do not significantly influence fertility attitude. To specify, people who considered the opinions of their peers more seriously tend to have a lower fertility intention. The reason might lie in that the young generation in China might consider the responsibility to have a child as an old-fashioned idea [25]. Similarly, fertility attitude was significantly associated with perceived behavioral control of finance while it was marginally related to perceived behavioral control of energy. People who had more monetary resources tended to have a stronger intention to have children, which is also consistent with previous literature [58,59].

Moreover, age was also a reliable indicator of fertility intention: older people tended to give up having children more easily. Gender tends to work as a marginal factor for fertility intention: compared with men’s fertility intention, women showed a marginally weaker intention to have children. That might be caused by Chinese women’s increased education level and work participation [71].

## 6. Discussions

This study tended to have the following theoretical contributions. To begin with, although some prior research tried to address the antecedents of fertility intention and birth-intervention policies in the context of China [25,58,59,71,72], they largely neglected to address this issue by introducing the latest technology applications, an emerging trend for adopting baby robots to encourage people’s fertility intention. By exploring the relationship of UV effect in baby robots and fertility intention, the current research examines an emerging way to increase people’s fertility attitude, thus promoting fertility intention at the end. Furthermore, previous research on the theory of planned behavior has treated the subjective norms and perceived behavioral control as a single construct. Based on the decomposed theory of planned behavior, the current study subdivided subjective norms and perceived behavioral control into sub-constructs and explored their influence on fertility intention in the context of China, trying to achieve a detailed picture of Chinese fertility behavior.

This study also has some limitations. First, this research explored the relationship between the uncanny valley and people’s fertility intentions, providing the preliminary guidelines for robot designers. The robot No.6 was adapted from a real infant picture which by far cannot be made at the current state of the robot industry. Thus, it might work as a confounding factor since people might be biased due to its realistic appearance. Future studies should try to use different actual social robots to validate the current result.

Second, there are many other features of a social robot that could be explored and work as promoting factors for people’s fertility intention, such as specific facial features [73], expression [74], and related other traits, which future study should try to explore.

Third, the main goal of this study was to explore the effect of the baby robot on fertility intention. Though this study recruited sufficient participants to take part in the experiment, the sample size might be relatively small when exploring the relationship between fertility attitude, subjective norms, perceived behavioral control, and fertility intention. Future studies should try to introduce a sizeable domestic survey to validate the conclusions in the current study.

Fourth, the measurement of behavioral control and fertility intention might be limited. The current study did not differentiate in the difference between the extensive and intensive margin of fertility, which might work as a cofounding factor. Further studies should try to examine the factors in detail and validate the current finding. Though the current study adapted the items from two perspectives, namely finance and physical energy [54,55], there might be more emerging factors to influence people’s fertility ability, such as parenting skills [75] and related attitudinal factors [76,77], which could be included in future studies. 

## Figures and Tables

**Table 1:** The measurement items and relevant definitions.

Measure	Definition	Measure Items
Fertility Attitude	It refers to an individual’s evaluation of the extent to which one is in favor or not of fertility behavior.	I would consider the bond between husband and wife when deciding to have a child.
Subjective Norms	It refers to social pressure one feels while deciding whether to implement certain behaviors.	I would consider the opinions of my relatives when deciding to have a child. The opinions of my friends could influence my decision on childbearing.
Perceived Behavioral Control	It refers to the extent of individual control which one perceives that he/she has of his/her fertility behavior.	I feel I have the energy to handle taking care of a child. I feel I can get enough financial resources to raise a child.
Fertility Intention	It is defined as the imagination and longing of husband and wife for their own family	The difference between the ideal and actual number of children in the survey.

**Table 2:** Demographic characteristics of the sample.

Attributes	Value	Frequency	Percentage (%)
Gender	Male	101	56.10%
	Female	79	43.90%
Age	20–24	14	7.78%
	25–29	55	30.56%
	30–34	37	20.56%
	35–40	37	20.56%
	40–	37	20.56%
Education	High school or below	30	16.67%
	Undergraduate or above	150	83.33%
Marital	Married	174	96.67%
	Divorced, separated or widowed	6	3.33%

**Table 3:** Distribution of the difference between the ideal and actual number of children.

Ideal Number	Actual Number				
	None	One	Two	Three	Total
None	34	0	0	2	36
One	22	22	4	0	48
Two	20	15	31	4	70
Three	6	6	8	6	26
Total	82	43	43	12	180

**Table 4:** The mean of different variables and their correlations.

Construct	Mean	SD	FA	SF	SP	BF	BE
Parenting Attitude (PA)	6.41	2.36	1				
Subjective Norm-Family (SF)	4.52	2.49	0.326 ***	1			
Subjective Norm-Peers (SP)	4.32	2.51	0.237 ***	0.792 ***	1		
Behavioral Control-Finance (BF)	6.59	2.04	0.542 ***	0.079	0.045	1	
Behavioral Control-Energy (BE)	6.36	2.06	0.445 ***	0.219 ***	0.201 ***	0.479 ***	1

Note: *** *p* < 0.01.

**Table 5:** The mean, SD, *F* value, *P* value of different variables and their ANOVA.

Constructs	No.1 Robot Mean (SD)	No.2 Robot Mean (SD)	No.3 Robot Mean (SD)	No.4 Robot Mean (SD)	No.5 Robot Mean (SD)	No.6 Robot Mean (SD)	*F* value	*P* value
Fertility Attitude	6.80 (2.06)	6.83 (2.55)	4.90 (2.92)	5.70 (2.26)	7.03 (1.77)	7.17 (1.64)	4.86	*p* < 0.05
Subjective Norm-Family (SF)	4.17 (2.30)	4.02 (2.68)	4.41 (2.86)	4.77 (2.43)	4.89 (2.26)	4.90 (2.35)	0.73	ns
Subjective Norm-Peers (SP)	4.07 (2.41)	4.2 (2.59)	4.17 (2.83)	4.43 (2.46)	4.47 (2.37)	4.61 (2.52)	0.20	ns
Behavioral Control-Finance (BF)	6.50 (1.79)	7.07 (1.96)	6.02 (2.42)	6.57 (2.14)	6.33 (2.02)	7.07 (1.76)	1.27	ns
Behavioral Control-Energy (BE)	6.63 (1.69)	6.93 (1.94)	5.71 (2.49)	5.90 (2.18)	6.03 (1.99)	6.97 (1.75)	2.22	ns

**Table 6:** The logistic regression on fertility intention.

Constructs	Sub-constructs	Coefficient	Odds Ratio
**Parenting Attitude**		0.238 **	1.269
**Subjective Norms**	Norm-Family	0.65	1.067
	Norm-Peers	−0.231 **	0.794
**Perceived Behavioral Control**	Control-Finance	0.253 **	1.288
	Control-Energy	−0.196 *	0.822
**Demographics**	Gender	−0.610 *	0.544
	Age	−0.060 ***	0.942
	Education	0.631	1.880

Note: * *p* < 0.10; ** *p* < 0.05; *** *p* < 0.01. Nagelkerke *R*-square: 0.215; Identification Accuracy: 68.9%.
